# Saskatoon, Saskatchewan’s safe consumption site provides a sizeable social and economic return on investment

**DOI:** 10.3389/fpubh.2026.1783089

**Published:** 2026-09-14

**Authors:** Linzi Williamson, Anne Miller, Maryellen Gibson, Maggie Coupland, Evan Poncelet, Kayla DeMong, James Dixon, Barbara Fornssler

**Affiliations:** 1 University of Saskatchewan, Saskatoon, SK, Canada; 2 Constellation Consulting Group, Calgary, Alberta, Canada; 3 Prairie Harm Reduction, Saskatoon, SK, Canada

**Keywords:** harm reduction, safe consumption, social return on investment, social value, substance use

## Abstract

**Introduction:**

The relative social value of safe consumption sites (SCS) in Canada is hotly debated, with sites facing community, media, and political backlash. Despite attempts to highlight community need and benefits of SCS, funding denials and site closures have repeatedly occurred.

**Methods:**

The objective of the current study was to highlight the social and economic value of Saskatoon, Saskatchewan’s SCS for relevant groups, including people who access services (PWAS), loved ones of PWAS, the broader community, business owners, and government systems and service (e.g., hospitals, police). This was achieved by conducting a social return on investment (SROI) using Social Value International’s framework. Social value was calculated by assigning financial proxies to represent the social, economic, and environmental value created by each outcome for each of the material groups, adding these values together, factoring in counterfactual variables, and dividing this number by the yearly operational costs of the SCS. Outcomes were determined via participant interviews (*N* = 26) and reviewing SCS administrative data. To avoid overclaiming, the study took a conservative approach whereby the lowest financial valuation options were assigned to each outcome and negative outcomes for each relevant group were accounted for.

**Results:**

The study found that for every dollar invested in the SCS operations, an estimated $4.44 CAD in social and economic value is created for all material groups.

**Discussion:**

Overall, the study demonstrated that Saskatoon, Saskatchewan’s SCS provides a sizeable social and economic return on investment for several relevant groups.

## Introduction

Canada’s overdose crisis has been ongoing for nearly a decade, resulting in over 52,544 deaths, 48,108 hospitalizations, 198,811 emergency department visits, and 244,927 emergency medical service responses to suspected overdoses (1). The synthetic opioid fentanyl and its analogues in the unregulated drug supply have been responsible for the majority of overdose deaths, with 74% of apparent opioid toxicity deaths involving the substance in 2024 (1). Opioids accounted for 14.4%, or $7.1 billion CAD, of the total costs of substance use in Canada between 2007 and 2020 (2). This crisis is driven by complex factors, including contaminated drug supply, prescription opioids, and opioid tolerance and dependence (3). Strategies employed to address this crisis are increased border control to stop the flow of illegal fentanyl, improving access to trauma-informed, culturally grounded, community-based mental health, and various substance use services including harm reduction initiatives, educational campaigns, and training and distribution of naloxone and overdose prevention materials (1). Supervised consumption sites (SCS) are another available strategy that spans substance use prevention to treatment (4). SCS, sometimes also referred to as supervised injection facilities when services are limited to supporting safer injection use (5, 6) or overdose prevention sites (7), are designed to offer safe, non-judgmental spaces for people who use drugs where they can consume substances they have obtained elsewhere under the supervision of medical care providers and individuals trained in administering Naloxone (brand name, Narcan) (8, 9). Additionally, sterile equipment can be accessed at many of these sites, which can minimize infection risks and secondary health concerns, and people can be referred to additional essential health and social services (e.g., housing support, treatment) (8). As of September 2024, there were 39 SCS operating in Canada (10). However, Ontario, for example, has seen recent closures of SCS locations as of March 31, 2025 with the passing of the Community Care and Recovery Act, 2024 (11). While criticisms of SCS often center around ideas that they “enable drug use and crime,” peer-reviewed research consistently shows this is not the case (12–14). Results of systematic reviews synthesizing studies from across the globe have found that SCS mitigate drug poisoning-related harm and unsafe substance use practices, facilitate uptake of substance use treatment and other health services, are associated with improvements in public order, do not increase drug-related crime, and are cost-effective (12–14).

Prairie Harm Reduction (formerly AIDS Saskatoon) in Saskatoon, Saskatchewan broadly offered services to individuals at high risk for and affected by HIV and Hepatitis C, provided drop- in outreach support, educational programs, and resources on various topics, and Needle Patrollers who picked up needles in key city neighborhoods. In October 2020, Prairie Harm Reduction opened Saskatchewan’s first SCS, following the S56.1 exemption to the Controlled Drugs and Substances Act approved by Health Canada and located adjacent to Prairie Harm Reduction’s drop-in center. The SCS offered low-barrier access for supervised substance use (i.e., inhalation room for smoking/vaping and booths for use of other substances), harm reduction education and supplies, assessments, referrals, and support services, and community awareness and drug alerts. Since opening, Prairie Harm Reduction’s SCS was consistently denied funding so operating costs were raised solely through fundraising or donation efforts (15). An economic assessment of Prairie Harm Reduction’s SCS has never been conducted.

Studies examining the cost-effectiveness and cost-efficiency of SCS (e.g., Canada, United States, France) have shown potential savings in public health expenditures and other benefits including saving lives and preventing infectious disease (5, 16–23). However, each region is unique with different substance use cultures which requires its own assessment. To complement the existing evidence base, better understand the social and economic value and impact of Prairie Harm Reduction’s SCS, and determine appropriate evaluation standards for this structural health intervention, a team of researchers, evaluators, and social value practitioners from the University of Saskatchewan undertook an evaluation and social return on investment (SROI) analysis. SROI analysis is a participatory evaluation approach that assesses the social, economic, and/or environmental value of change created for individuals who access or are affected by programs or services compared to the cost of investment in these programs or services (24, 25). SROI as a social and economic valuation method has not been previously applied to any Canadian SCS. However, SROI has been applied in a related Canadian context focused specifically on a national virtual/mobile overdose response program (26). The purpose of the current exploratory case study was to determine the social and economic value of Prairie Harm Reduction’s SCS using Social Value International’s SROI methodology.

## Materials and methods

The project, titled *Building Capacity to Reduce Substance Use Harms: Researching Effective Evaluation Standards for the Supervised Consumption Site in Saskatoon SK* was funded with a two-year Solutions Impact Grant from the Saskatchewan Health Research Foundation (SHRF), in partnership with Prairie Harm Reduction, and with guidance from a First Nations and Métis Advisory Committee. A formative process-outcome evaluation was undertaken to assess the process of implementing and accessing the SCS, the extent to which the intended outcomes of the SCS were being achieved, and areas where the SCS could be adapted and improved for future use (27). In addition to ensuring a program’s effectiveness and impact, evaluation allows policymakers and program administrators to determine whether intended outcomes are being achieved and resources are efficiently used (28). The study evaluation was paired with a forecast SROI analysis, specifically, which estimates the amount of social, economic, and environmental value, a project or activity will create if the intended outcomes for target individuals/populations are met (29). This publication focuses on the SROI component of the project while the evaluation is indicated in a technical report (27).

### SROI participants

Ethical approval for this study was granted by the authors’ institutional research ethics board (#4161, September 8, 2023). SROI participants were identified through a combination of trusted relationships, purposive sampling, and convenience sampling—people who access services (PWAS, referred to here on out as service users) were invited by Prairie Harm Reduction based on site engagement, external stakeholders were selected by the project team for their organizational relevance to the SCS, and SCS staff were interviewed based on availability during site visits. For the SROI calculation, online groups deemed “material” were included. Borrowed from the world of accounting, groups or information are “material” if they affect readers’ or decision-makers’ decisions or if the exclusion of the groups or information would misrepresent an organization’s activities (27). Materiality for this evaluation was determined in consultation with Prairie Harm Reduction, the First Nations and Métis Advisory Committee, and consulting SROI best-practice literature before being validated during data collection interviews. The names of all participants were changed to protect their identity.

### Program logic model and theory of change

The program logic model (PLM) (Figure 1) and theory of change (ToC) for Prairie Harm Reduction’s SCS were initially developed by two of the research team’s Credentialed Evaluators (CEs) based on a review of program documents, a research literature review, and engagement with Prairie Harm Reduction staff. The PLM was then confirmed and revised based on participant interviews and organizational documents review (described below). The PLM provides a visual depiction of the underlying ToC, which is a description of how resources (inputs) are used to deliver activities (outputs) that result in changes (outcomes) for target group(s) (participants). These are essential for the SROI analysis as this information is used to determine components such as relevant outcomes, additional material groups, and counterfactuals (described in the Results). A detailed explanation of the PLM and underlying ToC can be accessed in the accompanying evaluation report (27). Given the complexity of the PLM, it is not possible to detail every ToC in this publication. One of the simplified ToCs for people who use drugs (PWUD), for example, is as follows: access to supervised drug consumption leads to prevention of adverse health outcomes like overdose then changes in behavior like identification of and movement toward substance use recovery goals then in the long-term improved overall health and wellbeing.

**Figure 1:**
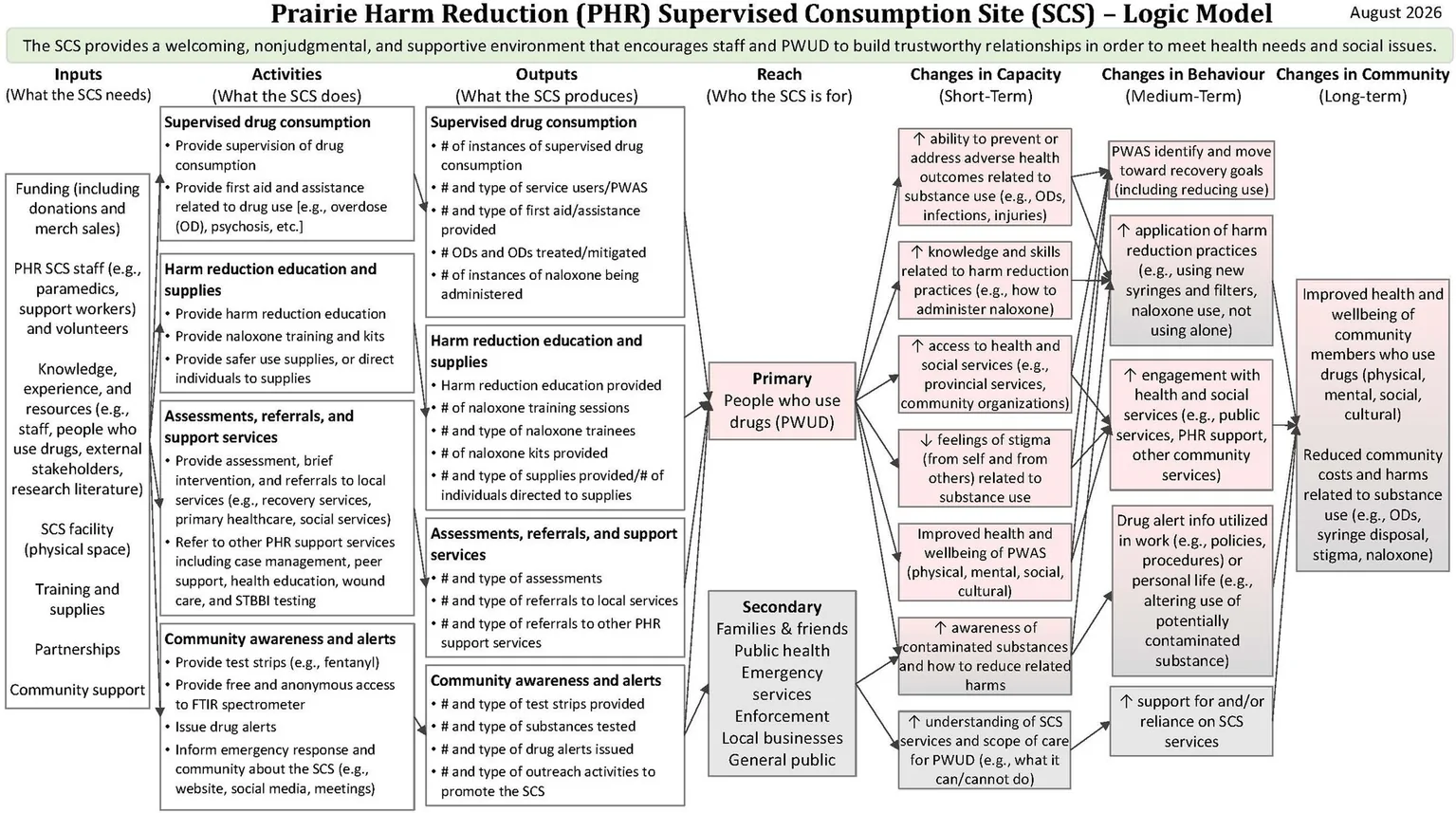
Program Logic Model for Prairie Harm Reduction’s Safe Consumption Site.

### Interviews

Semi-structured interviews (*N* = 26) were completed with *n* = 10 regularly and/or recently attending service users between June 23, 2023 to July 3, 2023, *n* = 6 Prairie Harm Reduction staff members between November 16, 2023 to January 17, 2024, and *n* = 10 representatives from external organizations that interact with and/or have an interest in the SCS, including members of primary care services, emergency services, community-based organizations, and public health, between September 18, 2023 to December 18, 2023. The purpose of the interviews were to confirm and add relevant components to the initially drafted PLM and derive information to complete the SROI (e.g., relevant outcomes, external factors, etc.). Interview questions were developed in consultation with Prairie Harm Reduction representatives (e.g., Executive Director, Staff) who collaborated with the research team. The questions for each participant group (i.e., service users, staff, and external representatives) differed slightly based on the kind of information they could access and provide (Table 1). Service users and staff interviews took place at the adjacent Prairie Harm Reduction drop-in center in a private room and external interviews took place online via Zoom. Two research team members, who have experience working with SCS staff and service users, conducted the interviews. Service user recruitment was overseen by Prairie Harm Reduction given their trusting relationship. Interviewed staff included an employed paramedic and other staff who were available when the interviewers visited the SCS. External interviewees were purposively selected by the research team who chose representatives from a range of organizations and sectors who interact or share additional support services with individuals who also access the SCS. These interviews occurred online via Zoom. Service user interviews ranged from 51 to 70 min (*M* = 27.8), staff interviews 15–41 min (*M* = 33), and external representatives 33–78 min (*M* = 51.4). Audio recordings of interviews were transcribed by research team members and contracted graduate students. Data were analyzed by two CEs (authors 1 and 5) and using Braun and Clarke’s guide for thematic analysis (30). Deductive coding (i.e., use of a pre-established coding framework) was used when confirming aspects of the PLM, otherwise inductive (i.e., open) coding was applied. After initially coding one of each type of participant interview, the CEs met to review and discuss. Following this, author 1 who is also a social value practitioner completed coding for all aspects of the SROI while author 5 completed coding for the PLM. Once coding was complete, codes were then organized into preliminary themes, discussed with the group, and then finalized.

**Table 1:** Participant interview guide.


Service users interview questions
Overall, are you happy with the supervised consumption site (SCS)? What are you most happy with? What are you least happy with? Is there anything you’d like to be done differently? Have you accessed supervised consumption or similar programming from other organizations, recently, or in the past? How were those programs similar/different to PHR’s? We will now review the drafted program logic model, which is an image that shows how a program works, for Prairie Harm Reduction’s (PHRs) supervised consumption site (SCS): Prior to this interview, we determined some key changes that can happen for folks who access the SCS, also known as outcomes, in conversation with other folks accessing services and PHR staff, and we would like to if any of these [refer to drafted logic model] occurred for you: Short-term outcomes/changes Intermediate-term outcomes/changes Long-term outcomes/changes Are there other programs or organizations that have helped you with any of these outcomes/changes? Has your life changed since accessing the supervised consumption site? How so? What do you do differently now? Next, we will examine some information that will help us conduct the social return on investment: How valuable have the outcomes/changes you described been for you? How valuable have the outcomes/changes been for your family? Your community? If you had to put a price or dollar amount on them, what amount would you choose? Beyond putting a dollar amount on the outcomes/changes, what are some of the other ways the program has added value to your life? Approximately, how long do you experience the outcomes/changes? What would you do if you could not access the supervised consumption site anymore? What would you do differently? Where would you go? Are there any associated costs with this? Economic, personal, legal, social? What do you think are some examples of dollar estimates or values, also known as financial proxies, for the outcomes/changes you have experienced because of accessing the SCS? Is there anything else you’d like to share with us today about your experience with the SCS?
SCS staff and external stakeholder interview questions
We will begin by reviewing the drafted program logic model for Prairie Harm Reduction’s (PHRs) supervised consumption site (SCS): What are the program inputs? What is needed to run the SCS? What is the approximate cost for each of the program inputs? What are the program activities? What does the SCS offer people accessing its services? Who are the program activities intended for (i.e., target groups)? What are the outputs of the activities of the SCS? What activities do you measure or collect data on? E.g. number of Narcan kits used What are the assumptions of the program? What can we assume is happening for the activities to occur as intended (e.g., people have to be engaged with the activity)? What short-term outcomes/changes can you expect to see for the target groups? For example, can you expect to see cognitive/mental or other immediate changes? What intermediate-term outcomes/changes can you expect to see for the target groups? For example, can you expect to see behavioral changes? Or changes that are the next logical step from the short-term outcomes/changes? What long-term outcomes/changes can you expect to see for the target groups? For example, can you expect to see community- or social-level changes? Or changes that are the next logical step from the intermediate-term outcomes/changes? What are some potential indicators of the short-, intermediate-, and long-term outcomes/changes that we could feasibly measure? Next, we will examine some information that will help us conduct the social return on investment: Are there examples of dollar estimates or values, also known as financial proxies, you can identify for each of the outcomes/changes? Are there any examples of sources of deadweight (i.e., aspects of change that would have happened anyway or are the result of other factors) and/or attribution (i.e., how much of the outcomes was caused by the contribution of other organizations or people) you can identify for us? Are there any unintended outcomes/changes we can expect because of the activities? What do you think is the estimated duration of each outcome/change? How long to expect effects to last for target groups? What are the external risks to the program (i.e., what may negatively affect the program)? For example, removal or decreased government funding? What do you see as the social value of the program and outcomes/changes for target groups? Where do you expect to see value? E.g. economic (employment, cost effectiveness/savings), public safety and legal (community safety, policing and crime, corrections), healthcare (hospital, emergency medical services, illness and death) Is there anything else you’d like to share with us?

### Documents review

Administrative data collected by the SCS between May 2023 and April 2024, including client intake information and drug checking (i.e., test strips, spectrometer results) were reviewed. Like the interviews, the purpose of the documents review was to confirm and add relevant components to the initially drafted PLM and derive information to complete the SROI (e.g., relevant outcomes, frequencies, etc.).

### SROI analysis and modeling

This study attempted to explore the value of outcomes during participant interviews using willingness to pay, willingness to accept, and stated preference metrics (31, 32). Interviewed service users were asked “What are the most valuable changes that have occurred for you as a result of your experience with Prairie Harm Reduction’s SCS?.” Interviewed service users, however, found it difficult to apply values to outcomes. Ethically, and by direction of Prairie Harm Reduction’s Executive Director, service users were not directly asked about the financial value of some outcomes, chiefly those pertaining to the preservation of human life, to avoid inducing substantial mental and emotional harm. As such, desktop research (i.e., searching peer-reviewed and gray literature) was undertaken and revealed preference metrics were applied by the two SROI practitioners on the team to identify suitable financial proxies for each outcome and determine appropriate discount factors (i.e., deadweight, displacement, benefit period, drop off).

The value of program inputs for the May 2023 to April 2024 period when the evaluation and SROI occurred was provided by Prairie Harm Reduction’s Executive Director based off financial records of yearly estimated costs for operating the SCS. The social value of Prairie Harm Reduction’s SCS was calculated using the internationally standardized six-step SROI methodology as outlined in *A Guide to Social Return on Investment*, published by Social Value International (25). The SROI methodology provides a framework for articulating the financial value of social outcomes created through key investments, revealing how much value is created for every dollar invested. SROI goes beyond standard cost–benefit analysis by focusing on the value of outcomes experienced by a variety of stakeholders, rather than focusing solely on investments and outputs. This means that social outcomes, such as increased wellbeing, are represented in financial terms alongside more tangible cost savings and value creation for governments, communities, and individuals. While SROI enables analysis of the value of social outcomes using the language of financial returns, the social and economic value return calculated through an SROI analysis is not equivalent to a financial return in spendable dollars. It is better understood as an approach to valuing social outcomes through financial measures other than standard economic indicators, such as GDP (see Ravi and Reinhardt (33) and Siglitz (34)). Outcomes included in the SROI analysis were based on outcomes included in the PLM, participant interviews, documents review, as well as outcomes highlighted in published literature and outcomes highlighted by the local community (e.g., changes in community safety and business opportunities articulated in local news reports). The value of each outcome was then determined, with financial proxies used to represent the social, economic, and environmental value created by each outcome for each of the groups who were deemed material when direct financial value was not possible to capture (see Results below).

Percentage discounts were then applied for each outcome (direct or represented using a financial proxy): deadweight, attribution, displacement, benefit period, and drop-off. Deadweight—a percentage of the amount of outcome that would have occurred even if the activity had not taken place (25)—was challenging for interviewed participants to estimate. As such, the SROI practitioners (authors 1 and 2) applied deadweight estimates ranging from 0% to 30% for all outcomes based on community and research evidence that the outcomes might otherwise have happened without the existence of the SCS (e.g., the existence of other supports that could have supported service users in achieving the same outcomes). A range of 0%–30% aligns with other SROI studies [see, e.g., Miller and colleagues (35), Pham and Kalagnanam (36), and Rioux and colleagues (26)] where observed outcomes are strongly linked to the services provided, while acknowledging that some outcomes may also be influenced by other factors. SCS provide direct and evidence-based interventions that reduce overdose deaths, improve access to healthcare, connect individuals to treatment and social services, and decrease public drug use. As a result, many outcomes can reasonably be attributed largely to the intervention itself, resulting in relatively low discount rates (0% in many cases). However, clients may also access support from healthcare providers, housing services, income assistance programs, community organizations, family members, or peer networks, all of which may contribute to observed changes. Applying discounts of up to 30% helps account for these external influences and the uncertainty inherent in assigning value to complex social outcomes. The selected range reflects a balanced and conservative approach, recognizing both the significant contribution of the safe consumption site and the broader network of supports that may influence client outcomes. Specifically, outcomes with 0% deadweight applied were related to instances whereby the outcome could not be achieved without access to SCS services and would be unlikely to occur without support. Responses from external interviewees and SCS staff support this: “*Prairie Harm Reduction is providing a service in the community unlike any other place (supervised consumption)*” (External interviewee P10) and “*Nobody else is doing any of that work—there’s no drug checking, no consumption site*” (Staff interviewee P6).

Attribution—a percentage estimate of how much of the outcome was caused by other organizations or people (25)—was considered for all outcomes. All participants acknowledged the intertwined nature of Prairie Harm Reduction’s services and the SCS. As such, an attribution of 50% was applied to many outcomes. In cases where the SCS provides unique, and non-stigmatizing, services (e.g., access to naloxone and oxygen to reverse overdoses, needle exchanges, wound care, drug testing), a 0%–10% estimate of attribution to other sources was applied by the SROI practitioners. When services were considered widely available elsewhere (e.g., STBBI testing) or not available at Prairie Harm Reduction or the SCS (e.g., family and relationship counseling) a 75%–90% estimate of attribution was applied. Interviewed participants acknowledged that the SCS cannot provide everything that people need, but noted that it was an important service and entry point:

“*…you cannot get everyone to do everything. So, I see it as an entry point that can be leveraged to do more things say if they wanted to in the future…I find like [service users] prefer community resources rather than going to like acute care or even like certain walk-in clinics and stuff like people will try to use services that they are most familiar and safe with to do things that, they get lots of overlap between them.*” – External P1.

Displacement is a percentage estimate of how much an activity has displaced other positive outcomes (25). Interviewed participants were unable to provide estimates of displacement. In cases where negative outcomes were accounted for and valued within the SROI analysis, a displacement of 0% was applied by the SROI practitioners. Some of the negative outcomes accounted for in the SROI analysis and valued with negative-value financial proxies included: service users experiencing increased disconnection with loved ones due to their continued substance use, service users increasing or altering their substance use, perceived decreased community safety, increased local public substance use when the SCS is closed, local businesses experiencing decreased foot traffic and decreased revenue, local businesses experiencing increased nuisance, increased emergency response to overdoses, and increased police call-outs related to public SU, social disorder, and violence. Outcomes where 10%–30% displacement was applied were: service users experiencing decreased substance use stigma and internalized stigma to account for increased stigma due to increased awareness of substance use due to the SCS; service users experiencing improved mental health and personal identity since service users may experience the opposite when accessing support for SU; service users experiencing improved cultural health since this is not a main service focus for the SCS; service users experiencing improved relationships because connection to family or others may results in negative outcomes (e.g., family are most likely to traffic individuals); service users experiencing an increased sense of safety and protection since there may be instances of increased violence in the area surrounding the SCS; service users experiencing decreased suicide risk to account for increased stigma due to increased awareness of substance use due to the SCS; and, loved ones of service users experiencing decreased stress/worry to account for possible increased stress/worry from ongoing substance use by service users.

Outcome duration or “benefit period” is the amount of time an outcome will last into the future without further investment or access to services (e.g., if the SCS closed, how long would outcomes included in the SROI analysis last?), and drop-off is the deterioration of an outcome over time (25). These elements were also challenging for interviewed participants to estimate, though when the benefit period does not extend into the future, then there is no possibility for drop-off of the benefit over time. In cases where outcomes may extend into the future even if the SCS did not exist (e.g., decreased community safety due to ongoing substance use in the area, despite closure of the SCS), a benefit period of 1 year was estimated along with a drop-off rate of 10%. For most outcomes, no duration of the outcome into the future was included in the SROI model, and a drop-off of 0% was thus applied to most outcomes. For any outcomes with duration into the future, a discount rate of 3.5% was applied to the future value of the outcome to account for the time-value of money.

The SROI ratio was calculated by multiplying the total value of each outcome by the number of people/stakeholders experiencing the outcome (i.e., frequency) and the duration of the benefit, where applicable. Discounts (deadweight, attribution, displacement, drop off) for each outcome value are then applied as percentage reductions to the total calculated value of the outcome. For example, if an outcome is valued at $10,000, with 20% deadweight and 10% attribution, the adjusted value would be: $10,000 × (1–0.20) × (1–0.10) = $7,200. Once all outcome values have been discounted, they are summed to calculate the total social value created. If the analysis covers multiple years, future benefits are also discounted to their present value using an appropriate discount rate, however, the current analysis did not include any future value of outcomes, so an overall discount rate was not applied to the analysis. The SROI ratio is then calculated by dividing the total present value of all discounted outcomes by the total value of the investment required to operate the safe consumption site:

$$\begin{array}{l} \text{SROI Ratio}=\\ \text{Total Present Value of}\;\mathrm{all}\;\text{Discounted Outcomes}/\text{Total Investment}. \end{array}$$

The results of the SROI analysis were presented to Prairie Harm Reduction staff, interview participants, and service users for validation and feedback before finalizing the model. Given the contentious nature of harm reduction and SCS services in Saskatoon area, the fact that there is still more to learn about value of the SCS’ impact on service users and the Saskatoon community, and because SROI is still evolving as a measurement framework, a conservative approach was adopted for decisions on data and assumptions used in the SROI calculation. Specifically, the lowest financial valuation options for each outcome were used, the minimum number of stakeholders reasonably expected or demonstrated to experience each outcome was used, and no future value was claimed (i.e., longer-term impact and resulting financial value were not included in the analysis in comparison to current year investment). As such, values in this report might understate the actual value created. In line with the conservative approach to valuing outcomes resulting from the SCES to avoid overclaiming, as discussed above, the study also accounted for negative outcomes for each material group.

Financial values and proxies were chosen based on alignment with outcomes and the local Saskatoon context, prioritizing inclusion of local values, Canadian values, and recent published values. Where values from years prior to the study year were used, they were adjusted for inflation using the Bank of Canada inflation calculator (37). Where values from outside Canada were used, World Bank purchasing power parity (PPP) adjustments based on the year of the valuation were applied to convert the financial value to equivalent Canadian dollars (PP is designed to account for differences in the cost of living and purchasing power between countries, rather than simply converting currencies at market exchange rates) (38). The equivalent Canadian dollar values were then adjusted for inflation using the Bank of Canada inflation calculator. Where values from outside Canada were used, only values from countries with similar historical and current context to Canada were used (e.g., Australia, United States, New Zealand). Service users found it difficult to place a financial value on outcomes they experienced for various reasons, including lack of experience and skill in establishing a price for outcomes that are not bought or sold in typical marketplaces (e.g., improved well-being), fear that they might undervalue intangible outcomes which could negatively impact future services, and ethical and moral concerns related to pricing outcomes related to mortality, health, and wellbeing.

Sensitivity tests were used to test assumptions about values or financial proxies included in the analysis. Sensitivity testing is conducted in an SROI analysis to assess how changes in key assumptions affect the estimated social value and resulting SROI ratio. The process involves systematically varying one or more assumptions while holding other variables constant and recalculating the SROI ratio to observe the impact on results. The resulting range of SROI ratios demonstrates how sensitive the findings are to changes in key inputs. If the SROI ratio remains positive and substantial under conservative assumptions, confidence in the results is strengthened. Conversely, if small changes in assumptions produce large changes in the ratio, the analysis may indicate greater uncertainty and highlight areas where additional evidence may be needed.

The SROI investigated outcomes for five groups who were determined to experience material outcomes/value: (1) service users; (2) residents of the Saskatoon community; (3) local business community; (4) loved ones of service users; and (5) government systems and services (e.g., hospitals, police). Service users were included because they are the primary beneficiaries of the SCS, experiencing direct benefits from use of SCS services. Residents of the Saskatoon community were included because they may be affected by and can impact the SCS services by way of voting and filing complaints. The local business community was included based on anecdotes that some local businesses are affected by SCS services. Loved ones of service users were included based on media and municipal reports from service users that they are impacted, albeit indirectly, by SCS services. Government services/systems were included as changes experienced by service users and other stakeholders can result in changes in government service/systems use.

## Results

There were numerous outcomes experienced by service users as evidenced by the participant interview data (see evaluation report (27) for more detailed results based on these interviews), documents review, and development of the PLM. Tables 2–6 present the list of outcomes for each material group. The outcomes rated most important to service users, in order from most to least, were: (1) keeping people alive (2) preventing overdoses and having the skills to do so (3) allowing people to connect with others, have a sense of community, and feel accepted in society (4) preventing adverse health outcomes like HIV (5) reducing substance use stigma, and (6) reducing burden on healthcare systems and related costs. According to the documents reviewed in this study, in the May 2023–April 2024 timeframe, the SCS reported 8,232 total visits from 866 unique service users (average of 686 visits/month) and tested 728 drug samples. Service users were between 30 and 59 years old, with a near even split of males (41%) and females (40%) and 18% not disclosing their assigned sex. Injection was the most common mode of substance consumption (71%) and the most used substances were hydromorphone/Dilaudid (39.1%), crystal methamphetamine (35.1%), and fentanyl (20.8%).

**Table 2:** Valuing outcomes experienced by SCS service users.

Outcome	Financial valuation or proxy	Value ($CAD)	Source	Notes on value included and sensitivity test conducted (if applicable)
# of avoided overdoses that might otherwise have resulted in death or serious harm.	Quality Adjusted Life Year (QALY) valuation.	$22,229	Lebenbaum et al. (52)	Adjusted for inflation from $18,853 in 2020. Selected for Canadian applicability, recentness of publication, and alignment with outcome. Sensitivity tested against higher value of a Statistical Life (VSL; Canada): 2009 article with 2007 cost estimates, $3.5–9.5 million (in 2025 = $5.1–13.9 million CAD adjusted for inflation) (53)
# of reversed overdoses resulting in avoided death or serious harm	Quality Adjusted Life Year (QALY) valuation	$22,229	Lebenbaum et al. (52)	As above.
# of substance use-related infections avoided (e.g., cellulitis, osteomyelitis)	Revealed preference valuation: Personal cost of being treated for cellulitis	$47	Cannon (54)	Direct cost of treating cellulitis at a hospital per person calculated as $50NZD in 2021. Converted to CAD and adjusted for inflation = $47.46 CAD in 2025. Selected based on comparability with Canadian context in the absence of Canadian valuation of this specific condition related to substance use
# of HIV infections avoided (minimum estimate of 1)	Quality Adjusted Life Year valuation	$22,229	Lebenbaum et al. (52)	As above
# of contaminated samples found	Cost of drug checking using Fourier-transform infrared spectroscopy and drug testing strips	$23,414	ScienceTech Shop online (55)	Based on the total number of substances checked with spectrometer (345) and the cost (in CAD) of spectroscopy machine, at the lower end are $13,799. UofT reports that hourly rates of use/testing at the university cost $17–22/h (CAD) while externally it can cost $29–66 CAD/h (https://analest.chem.utoronto.ca/rates), so we have got a range we can work with. Drug checking can be completed in about 10 min: https://www.fraserhealth.ca/Service-Directory/Services/mental-health-and-substance-use/drug-checking---Fourier-transform-infrared-spectrometers-ftir Other sources—Simple analyses can take a few hours to a day (https://www.frontiersin.org/journals/microbiology/articles/10.3389/fmicb.2025.1565888/full), but more complex ones can take several days: https://rockymountainlabs.com/how-long-does-ftir-analysis-take/
# of people avoiding physical harm (incl. death) due to weather-related dangers (e.g., frostbite, hypothermia, heat stroke, dehydration, etc.)	Revealed preference valuation: Cost of physiotherapy to address weather-related health issues	$110	Longevity Saskatoon Blog (56)	Value of physiotherapy to address weather-related health issues in 2025 specifically in Saskatoon. This is a revealed preference valuation financial proxy, indicating that the market value of the outcome can be represented by the cost others might pay to achieve the outcome (not implying that the service users in the study would pay for this outcome directly).
# of service users experiencing reduced stigma associated with substance use, increased self-acceptance/ decreased internalized stigma	Revealed preference valuation: cost of counseling to address internalized stigma (estimated at minimum, 2 sessions per month for 6 months)	$2,400	McCarty Counseling Individual Therapy Session Cost (57)	Value of counseling to address internalized stigma estimated at 2 sessions per month for 6 months at $200 per session specifically in Saskatoon in 2025. This is a revealed preference valuation financial proxy, indicating that the market value of the outcome can be represented by the cost others might pay to achieve the outcome (not implying that the service users in the study would pay for this outcome directly).
# of service users experiencing improved mental health and personal identity (e.g., Increased sense of pride, self-worth, purpose, autonomy, hope, personal growth, empowerment, trust, and feeling valued, accepted, validated, understood, not judged, welcome, dignified, treated with compassion, respected, decreased stress and worry); Decreased mental health disorder symptoms (e.g., decreased anxiety and depression)	Revealed preference valuation: cost of a life coach (minimum 3 sessions)	$150	Superprof Blog on Life Coaching (58)	Value of using a life coach (min 3 sessions) to work on personal identity issues, specifically in Saskatoon in 2025. This is a revealed preference valuation financial proxy, indicating that the market value of the outcome can be represented by the cost others might pay to achieve the outcome (not implying that the service users in the study would pay for this outcome directly).
# of service users experiencing improved cultural health	Revealed preference valuation: Cost of one-night at cultural experience resort	$159	Dakota Du nes Resort Rate (47)	Value of a cultural experience to advance cultural health near Saskatoon in 2025. Estimated at 1 room for 1 adult for 1 night = $159 CAD. This is a revealed preference valuation financial proxy, indicating that the market value of the outcome can be represented by the cost others might pay to achieve the outcome (not implying that the service users in the study would pay for this outcome directly).
# of service users experiencing improved relationships (e.g., increased sense of home, family, community and increased relationship building/maintenance)	Revealed preference valuation: cost of counseling to work on relationships (estimated at minimum, 2 sessions per month for 6 months)	$2,400	McCarty Counseling Individual Therapy Session Cost (57)	As above.
# of service users experiencing increased disconnection with loved ones (e.g., service users accessing SCS when loved ones prefer they enter a treatment center or work toward sobriety)	Revealed preference valuation: cost of counseling (estimated at minimum, 2 sessions per month for 6 months)	−$2,400	McCarty Counseling Individual Therapy Session Cost (57)	As above.
# of service users experiencing increased sense of safety and protection	Personal cost of pain and suffering due to assault	$13,773	Zhang and Department of Justice Canada (59).	Adjusted for inflation from $9,547 CAD in 2008. While this is an older source, no more recent estimations of the cost to victims of pain and suffering in Canada were found.
# of service users experiencing decreased suicide risk	Quality Adjusted Life Year (QALY) valuation	$22,229	Lebenbaum et al. (52)	As above.
# of service users with reduced or altered substance use	Personal cost of supporting an addiction	$18,783	DeRiviere (60)	Adjusted for inflation from $12,617 in 2006. While this is an older source, no more recent estimations of the comprehensive personal cost of supporting an addiction in Canada were found.
# of service users with increased or altered substance use due to exposure and access to other service users and substances	Personal cost of supporting an addiction	−$18,783	DeRiviere (60)	As above.

Reported values were adjusted for inflation.

**Table 3:** Valuing outcomes experienced by Saskatoon Community.

Outcome	Financial valuation or proxy	Value (CAD)	Source	Notes on value included and sensitivity test conducted (if applicable)
# of community members with perceived increased community safety [e.g. reduced needle debris; decreased violence; decreased criminal activity (e.g., theft, vandalism)]	Value of neighborhood satisfaction (not too much nuisance in neighborhood, e.g., noise)	$221	Saville-Smith, Dwyer, and Warren (61)	Estimated value of neighborhood satisfaction valued at $190NZD converted = $155.12 CAD, adjusted for inflation in 2025 is $221.42 CAD. Selected based on comparability with Canadian context in the absence of Canadian valuation of this specific outcome.
# of community members with perceived decreased community safety [e.g., increased needle debris; increased violence; increased criminal activity (e.g., theft, vandalism)]	Value of neighborhood satisfaction (not too much nuisance in neighborhood, e.g., noise)	−$221	Saville-Smith, Dwyer, and Warren (61)	As above.
# of service users who decrease their public substance use (i.e., reduced exposure of community members to substance use, overdoses)	Personal annual cost of PTSD	$20,692	Davis et al. (62)	Converted from $19,630USD per individual with PTSD to CAD and adjusted for inflation. Selected based on comparability with Canadian context in the absence of Canadian valuation of this specific outcome.
# of service users who increase their local public use of substances when SCS closed	Personal annual cost of PTSD	−$20,692	Davis et al. (62)	As above.

Reported values were adjusted for inflation.

**Table 4:** Valuing outcomes experienced by local business community.

Outcome	Financial valuation or proxy	Value (CAD)	Source	Notes on value included and sensitivity test conducted (if applicable)
# of local businesses experiencing decreased foot traffic and decreased revenue as a result	Estimated at $100 reduced spending per month over 1 year	−$1,200	Estimate only	Due to a lack of published evidence, a minimum estimated value was used for this outcome
# of local businesses experiencing reduced nuisance	Revealed preference valuation: cost of a security system to monitor nuisance	$5,000	Security.org (63)	Value of additional security for businesses, specifically in Saskatoon in 2025. This is a revealed preference valuation financial proxy, indicating that the market value of the outcome can be represented by the cost others might pay to achieve the outcome (not implying that the all impacted businesses would pay for this outcome directly).
# of local businesses experiencing increased nuisance	Revealed preference valuation: cost of a security system to monitor nuisance	−$5,000	Security.org (63)	As above

**Table 5:** Valuing outcomes experienced by loved ones of service users.

Outcome	Financial valuation or proxy	Value (CAD)	Source	Notes on value included and sensitivity test conducted (if applicable)
# of loved ones of service users with reduced likelihood of losing loved one to overdose	Revealed preference valuation: cost of 4 h of personal counseling related to bereavement	$985	Psychologists’ Association of Alberta (64)	Estimated at 2 h of bereavement counseling per loved one per year. Nearest bereavement counseling cost. Adjusted for inflation to 2025 dollars. Valuation justification from: Pham. (2020) (36)
# of loved ones of service users with decreased stress/worry	The statistical value of a life year adjusted with disability weight for mild General Anxiety Depression	$2,912	Ravi, Siddiqi, and Sridhar (65)	Converted from $2,458 AUD in 2014 to CAD and adjusted for inflation. Selected based on comparability with Canadian context in the absence of Canadian valuation of this specific outcome for this specific population

Reported values were adjusted for inflation.

**Table 6:** Valuing outcomes experienced by government systems and services.

Outcome	Financial valuation or proxy	Value (CAD)	Source	Notes on value included and sensitivity test conducted (if applicable)
# of service users with decreased need for hospital/medical response to overdoses	Average cost of services provided to the typical inpatient adult (aged 18–59 years) experiencing a drug overdose in an acute care facility in Saskatchewan	$5,668	Estimated average cost of services provided to the average typical inpatient adult (aged 18–59 years) experiencing a drug overdose in an acute care facility in Saskatchewan in 2021–2022	Adjusted for inflation from $5,105 CAD per visit in 2021–2022, according to the Canadian Institute for Health Information’s Patient Cost Estimator (66)
# of service users with overdose addressed at SCS resulting in decreased emergency medical response to overdose.	Minimum cost of road ambulance in Saskatchewan	$360	Government of Saskatchewan (67)	As of 2025, the cost of basic call pick up rate ranges from $245 to $325 CAD for residents of Saskatchewan with a valid health card and $360 for non-residents of Saskatchewan
# of increased emergency medical response to overdose (e.g., people using in local area outside the SCS operating hours)	Minimum cost of road ambulance in Saskatchewan	−$360	Government of Saskatchewan (67)	As above
Decreased hospital visits due to weather-related issues (e.g., frostbite, heat stroke, etc.)	Average cost of services provided to the typical inpatient adult (aged 18–59 years) experiencing significant injury/exposure to elements in an acute care facility in Saskatchewan	$10,354	Estimated average cost of services provided to the average typical inpatient adult (aged 18–59 years) experiencing significant injury/exposure to elements in an acute care facility in Saskatchewan	Adjusted for inflation from $9,325 CAD per visit in 2021–2022, according to the Canadian Institute for Health Information’s Patient Cost Estimator (66)
# of service users with decreased hospital cost related to HIV infections in people who use drugs (minimum estimate of 1)	Hospital visit cost for person who uses injection drugs and has HIV in British Columbia	$5,985	Subedi et al. (68)	Adjusted for inflation and for Canadian applicability, recentness of publication, and alignment with outcome
Decreased police call-outs related to public substance use, social disorder, violence, etc.	Cost of police call for service (Canadian average)	$1,141	Public Safety Canada Community Safety and Countering Crime Branch, Research Division (69)	Adjusted for inflation and for Canadian applicability. While this is an older source, no more recent estimations of the comprehensive costs associated with a police call out were found
Increased police call-outs related to public substance use, social disorder, violence, etc.	Cost of police call for service (Canadian average)	−$1,141	Public Safety Canada Community Safety and Countering Crime Branch, Research Division (69)	As above

Reported values were adjusted for inflation.

### Valuing outcomes

Tables 2–6 also present the financial values (direct or represented by financial proxy) selected for each outcome included in the SROI analysis.

### The counterfactual—deadweight, attribution, displacement, benefit period, drop-off

Discounts applied to financial proxies for each material group are contained in Tables 7–11. The net social value was greatest for service users followed by the Saskatoon community at $3,805,328 and $1,008,357.22, respectively.

**Table 7:** Applied discounts—people who access services.

Outcome	Financial value (CAD)	Quantity	Deadweight	Attribution	Displacement	Benefit period (years)	Drop-off	Net social value or impact (CAD)
# of avoided overdoses that might otherwise have resulted in death or serious harm	$22,229	7	0%	10%	0%	0	0%	$140,045.54
# of reversed overdoses resulting in avoided death or serious harm.	$22,229	44	0%	10%	0%	0	0%	$880,286.22
# of substance use-related infections avoided (e.g., cellulitis, osteomyelitis)	$47	75	20%	0%	0%	0	0%	$2,847.60
# of HIV infections avoided (minimum estimate of 1)	$22,229	1	20%	75%	0%	0	0%	$4,445.89
# of contaminated samples found	$23,414	1	0%	0%	0%	0	0%	$23,414.00
# of people avoiding physical harm (incl. death) due to weather-related dangers (e.g., frostbite, hypothermia, heat stroke, dehydration, etc.)	$110	106	10%	50%	0%	0	0%	$5,247.00
# of service users experiencing reduced stigma associated with substance use, increased self-acceptance/decreased internalized stigma	$2,400	87	0%	30%	10%	0	0%	$131,544.00
# of service users experiencing improved mental health and personal identity (e.g., Increased sense of pride, self-worth, purpose, autonomy, hope, personal growth, empowerment, trust, and feeling valued, accepted, validated, understood, not judged, welcome, dignified, treated with compassion, respected, decreased stress and worry); Decreased mental health disorder symptoms (e.g., decreased anxiety and depression)	$150	346	20%	50%	10%	0	0%	$18,705.60
# of service users experiencing improved cultural health	$159	87	20%	50%	10%	0	0%	$4,956.98
# of service users experiencing improved relationships (e.g., increased sense of home, family, community and increased relationship building/maintenance)	$2,400	520	20%	90%	25%	0	0%	$75,822.40
# of service users experiencing increased disconnection with loved ones (e.g., service users accessing SCS when loved ones prefer they enter a treatment center or work toward sobriety)	−$2,400	87	20%	0%	0%	0	0%	−$166,272.00
# of service users experiencing	$13,773	87	20%	50%	30%	0	0%	$333,967.70
increased sense of safety and protection								
# of service users experiencing decreased suicide risk	$22,229	1	20%	50%	10%	0	0%	$8,002.44
# of service users with reduced or altered substance use	$18,783	260	20%	10%	0%	0	0%	$3,513,472.85
# of service users with increased or altered substance use due to exposure and access to other service users and substances	−$18,783	87	20%	10%	0%	0	0%	−$1,171,157.62
							Total	$3,805,328.60

**Table 8:** Applied discounts—Saskatoon community.

Outcome	Financial value ($CAD)	Quantity	Deadweight	Attribution	Displacement	Benefit period (years)	Drop-off	Net social value
# of community members with perceived increased community safety [e.g., reduced needle debris; decreased violence; decreased criminal activity (e.g., theft, vandalism)]	$221	25,868	0%	30%	0%	0	0%	$4,001,779.60
# of community members with perceived decreased community safety [e.g., increased needle debris; increased violence; increased criminal activity (e.g., theft, vandalism)]	−$221	25,868	10%	30%	0%	1	10%	−$3,601,601.64
# of service users who decrease their public substance use (i.e., reduced exposure of community members to substance use, overdoses)	$20,692	44	0%	10%	0%	0	0%	$819,403.20
# of service users who increase their local public use of substances when SCS closed	−$20,692	13	20%	0%	0%	1	10%	−$211,223.94

**Table 9:** Applied discounts—local business community.

Outcome	Financial value ($CAD)	Quantity	Deadweight	Attribution	Displacement	Benefit period (years)	Drop-off	Net social value
# of local businesses experiencing decreased foot traffic and decreased revenue as a result	−$1,200	10	10%	30%	0%	1	10%	−$7,560.00
# of local businesses experiencing reduced nuisance	$5,000	10	0%	30%	0%	0	0%	$35,000.00
# of local businesses experiencing increased nuisance	−$5,000	10	10%	30%	0%	1	10%	−$31,500.00
							Total	−$4,060.00

**Table 10:** Applied discounts—loved ones of service users.

Outcome	Financial value ($CAD)	Quantity	Deadweight	Attribution	Displacement	Benefit period (years)	Drop-off	Net social value
# of loved ones of service users with reduced likelihood of losing loved one to overdose	$985	51	0%	10%	0%	0	0%	$45,211.50
# of loved ones of service users with decreased stress/worry	$2,912	217	30%	50%	25%	0	0%	$165,492.60
							Total	$210,704.10

**Table 11:** Applied discounts—government systems and services.

Outcome	Financial value ($CAD)	Outcome incidence	Deadweight	Attribution	Displacement	Benefit period (years)	Drop-off	Net social value
# of service users with decreased need for hospital/medical response to overdoses	$5,668	1	0%	10%	0%	0	0%	$5,101.20
# of service users with overdose addressed at SCS resulting in decreased emergency medical response to overdose.	$360	51	0%	10%	0%	0	0%	$16,524.00
# of increased emergency medical response to overdose (e.g., people using in local area outside the SCS operating hours)	−$360	13	20%	0%	0%	1	10%	−$3,674.99
Decreased hospital visits due to weather-related issues (e.g., frostbite, heat stroke, etc.)	$10,354	106	10%	50%	0%	0	0%	$493,885.80
# of service users with decreased hospital cost related to HIV infections in people who use drugs (minimum estimate of 1)	$5,985	1	20%	75%	0%	0	0%	$1,197.00
Decreased police call-outs related to public substance use, social disorder, violence, etc.	$1,141	52	0%	30%	0%	0	0%	$41,532.40
Increased police call-outs related to public substance use, social disorder, violence, etc.	−$1,141	433	10%	30%	0%	1	10%	−$311,253.39
							Total	$243,312.02

### Program inputs

This study determined inputs for Prairie Harm Reduction’s SCS via consultation with the project advisory committee and through interviews with SCS staff and informants external to Prairie Harm Reduction. The financial resources required to operate the SCS with Prairie Harm Reduction’s Executive Director were confirmed. The operational costs of the SCS for May 1, 2023 to April 30, 2024 were valued at $278,512.92 CAD, which included administrative wages and benefits, program wages and benefits, accounting, client services, insurance and licenses, memberships, office supplies, and utilities.

### The social return on investment

The total present value of outcomes from the SCS between May 2023 and April 2024 was calculated as $1,236,734.96 after all outcomes were valued and discounts were applied. The operational costs for the SCS between May 2023 and April 2024 were $278,512.92. The resulting SROI calculation formula and SROI ratio are as follows:

$$\begin{array}{l} \text{SROI ratio for Prairie Harm}\;{\text{Reduction}}^{’}s\;\mathrm{SCS}\\ =(\text{\$}1,236,734.96)/(\text{\$}278,512.92)=4.44. \end{array}$$

The SROI ratio calculated through the current research indicates that for every dollar invested in the operation of the SCS, an estimated $4.44 in social and economic value is created. This value was experienced by all material groups. Sensitivity analyses were conducted on the developed SROI model to test different assumptions with the greatest potential influence on the final ratio and alternative scenarios (Table 12). These tests further affirm the conservative approach to calculating the final SROI ratio.

**Table 12:** SCS SROI sensitivity analyses.

Scenarios	Sensitivity analysis results
Scenario 1: Increasing the value of a human life proxy	A conservative proxy estimate of $22,229 QALY for the value of a human life was originally applied in the SROI model. However, a higher value of a statistical life (VSL) has been reported: $5,100,000 CAD, after adjusting for inflation (70). This increased the ratio from 4.44 to 851.49. Note: the 2007 VSL cost estimates reported by Chestnut and De Civita (70) ranged from $3.5 to 9.5 million CAD, which after being adjusted for inflation in 2025 increased to $5.1–13.9 million CAD. Our sensitivity analysis and subsequent ratio were calculated using the lower end of the reported range
Scenario 2: Increasing the value of a human life proxy and applying it to just one service user	The original model accounted for the number of service users who experienced the outcomes, based on available documentation. If the life of only one service user was helped, and the value of their life was estimated at $5,100,000 CAD, the ratio was 42.34
Scenario 3: Accounting for each outcome being experienced by just one person (e.g., service users, community member, etc.)	The original model accounted for the number of people (i.e., those deemed material) who experienced the outcomes, based on available documentation. When we accounted for only a single person experiencing each outcome, the ratio was 0.35
Scenario 4: Increasing the quantity of service users affected by 25%, reflecting an estimated increase in service access since 2023	The original model accounted for outcomes occurring between May 2023 to April 2024. Since then, there have been reported increases in service access and the number of service users who need support [e.g., (71)]. Increasing the quantity of service user experiencing each outcome by a conservative estimate of 25% resulted in the model increasing from 4.44 to 8.69
Scenario 5: Increasing the quantity of service users affected by 100%, reflecting an estimated increase in service access since 2023	Increasing the quantity of service users experiencing each outcome by 100%, which more closely reflects reported estimates [e.g., (71)] resulted in the model increasing from 4.44 to 21.51
Scenario 6: Increasing the quantity of service users, reflecting the number of reported overdoses in early 2025	In March 2025 alone, there were nine deaths and over 435 overdoses reported (72). Increasing the quantity of service users who avoided overdoses or had an overdose reversed to 435 increased the original ratio from 4.44 to 79.49

## Discussion

Even with a conservative approach, it was demonstrated with SROI that Prairie Harm Reduction’s SCS provided a sizeable social and economic return on investment. These findings align with previous studies that have demonstrated the cost-effectiveness and cost-efficiency of SCS in Canada and internationally as well as potential savings in public health expenditures (4, 5, 16–23). For example, Khair and colleagues (23) reported that the SCS in Calgary, Canada produced approximately $1,600 in cost savings for each managed overdose and savings of $2.3 million for the lifetime of the program. Jozaghi and Reid (20) determined that establishing SCS locations in Toronto would lead to an average benefit–cost ratio of 1–1.2 for the first two facilities and lead to cost savings of about $728,620 CAD per year based on the prevention of new HIV and hepatitis C infections alone. About 5 years prior to the opening of Saskatoon’s SCS, Jozaghi and Jackson (19) projected that the establishment of a single SCS would result in a 1–1.44 benefit–cost ratio, or $975,525 CAD in costs saved for the prevention of HIV, specifically, through a needle exchange program. In their systematic review of the potential value of establishing SCS locations across North America, Behrends and colleagues (16) found that across all included studies SCS were cost-saving or had favorable cost-effectiveness or cost–benefit ratios, particularly when considering overdose mortality outcomes and/or averting HIV and HCV infections. In France, cost savings and benefits were reported for SCS in Paris and Stasbourg in 2016 (17), specifically when accounting for HIV and HCV infections, skin abscesses and related infective endocarditis, drug overdoses, and emergency department visits. Direct comparisons of these past results with this study are challenging given the SROI method that was used, which has not been applied to an SCS context before, and because of the scope examined in the study. The reported SROI-specific ratio accounts not only for the prevention of infectious disease, overdose, skin/wound infections, and emergency department visits, but also several additional outcomes for different material groups including, but not limited to, connection with others, having a sense of community, feeling accepted in society, reducing substance use stigma, reducing burden on healthcare systems and related costs, and even negative outcomes such as local businesses experiencing decreased foot traffic.

In the present study, although the greatest value was demonstrated for service users and the broader Saskatoon community, there was an overall net positive value for loved ones of service users, the Saskatoon business community, and Saskatoon government systems and services. Consistent with previous evidence, this study demonstrates the importance of SCS as a harm reduction initiative in keeping people who use substances alive, preventing overdoses and having the skills to do so, offering a sense of community, reducing substance use stigma, preventing adverse health outcomes like HIV, and reducing burden on healthcare systems and related costs (4, 12–14, 16–23, 39). This study also demonstrates the value of using SROI methodology in this context, provides a comprehensive and quantifiable analysis of financial and non-financial outcomes for Prairie Harm Reduction’s SCS, and enables a more holistic understanding of its impact.

Since conducting this SROI on Prairie Harm Reduction’s SCS, rates of overdose in Saskatoon and the need for services increased. In March 2025, alone, Saskatoon firefighters responded to 509 overdose calls and April 2026 saw a record breaking 680 overdose calls, averaging 16 overdose calls per day (40, 41). On April 9, 2026, Prairie Harm Reduction ceased operations. Against the backdrop of shifting political investments, significant financial strain, and a dramatic increase in service demand, the organization was unable to maintain financial stability and was forced to close its doors. Currently in Saskatchewan, Nēwo-Yōtina Friendship Centre in Regina operates the province’s only SCS. Further, the Government of Saskatchewan has signaled a move toward a “recovery-oriented system of care” (ROSC) which has been coupled with the elimination of provincial funding for many harm reduction services and supplies including ending the distribution of safer smoking supplies and returning to a one for-one needle exchange policy (42). Alongside this policy change, the province has also classified hypodermic needles as street weapons, introduced substantial fines for fentanyl and methamphetamine distribution, and passed the Compassionate Intervention Act which seeks to mandate treatment for people who use substances (43–45).

### Strengths and limitations of the current study

While each Canadian city/region and SCS location is unique with different substance use culture and requires its own SROI assessment (28), thus limiting the generalizability of results of this study, there may be aspects of the current SROI that can be applied to other contexts (i.e., transferability) (46). In particular, if SCS services offered in other locations are similar to those offered by Prairie Harm Reduction’s SCS and service users and other material groups report experiencing similar outcomes because of the SCS, then transferability may be more feasible (47). The relatively small sample size of service users, staff, and representatives from external organizations connected to Prairie Harm Reduction may not have captured the full range of experiences and perspectives related to the SCS. However, the sample size is comparable to recommendations for quantitative studies and other published SROIs (48–50). Another limitation is that the interviewed sample in this study was not representative of all material groups, such as loved ones of service users, the broader Saskatoon community, the local business community, and government systems and services. These groups were not interviewed in the current study due to limited resources and because this was an initial exploratory case study. Although, outcomes for loved ones of service users, the broader Saskatoon community, the local business community, and government systems and services were included in this SROI after being identified by the included material groups and the team of this study upon review of news articles and empirical research and based on the developed logic model. Given the reliance on secondary/indirect information for these additional groups, there is an inherent limitation and bias to the results. Further, when interviewing service users, it was often difficult to elicit a detailed response in relation to costs attributable to not accessing the SCS or costs of certain interventions occurring at the site (i.e., overdoses reversed). Not only was it difficult to garner a response, the interviewers felt that asking such questions strained the rapport built with service users and negatively impacted the interview environment. It is the researchers’ opinion that while not gathering certain data from service users for the SROI may be considered a limitation of this study, the situation further highlights a potential limitation of the methodology when working with marginalized populations. Future research should consider how best to meaningfully engage with marginalized populations so that rapport and relationships are cherished and data relevant for research purposes is collected. When this project was unable to collect this specific data from service users, other sources were used. Future investigations may also benefit from including other relevant participant groups as there may be additional outcomes to consider. Given the interconnected nature of Prairie Harm Reduction’s drop-in center and the SCS site, it was difficult for some participants to isolate the unique impact of the SCS on service users and other relevant groups. The focus on only 1 year of SCS services limits the ability to forecast whether material group outcomes will persist over time. As such, future forecast SROIs on Prairie Harm Reduction’s SCS should include additional years and be monitored over time. Since there is inherent subjectivity in assigning monetary value to outcomes (51), future SROI investigations May yield different results. The decision of the team to take a conversative approach to selecting financial proxies for outcomes and ensuring all decisions were evidence-based was guided by this inherent limitation to SROI.

## Conclusion

Results of the current study complement previous evidence that Prairie Harm Reduction’s SCS saved lives, was an effective intervention for addressing Saskatoon’s overdose crisis, and generated social and economic value not only for the people who directly depend on services. Funding SCS is ethically, morally, and economically responsible and valuable. Next steps for this project are to present these findings and appeal for reliable and sustainable funding for the reopening of an SCS within Saskatoon and other communities within the province.

## Data Availability

The raw data supporting the conclusions of this article will be made available by the authors, without undue reservation.
